# Cardioprotective effect of genetic ablation of the G-protein-coupled receptor kinase GRK2 in adult pancreatic β-cells during high-fat diet

**DOI:** 10.1016/j.jbc.2025.108388

**Published:** 2025-03-05

**Authors:** Jonathan Snyder, Chun-sun Jiang, Ran Hee Choi, Taylor Morgan, Jeffrey Roman, Lilly Underwood, Anna Maria Lucchese, Sarah Montgomery, Laurel A. Grisanti, Nicolai Doliba, William L. Holland, Priscila Y. Sato

**Affiliations:** 1 Department of Pharmacology and Physiology, Drexel University, Philadelphia, Pennsylvania, USA; 2 Department of Medicine, Division of Cardiovascular Disease, University of Alabama at Birmingham, Birmingham, Alabama, USA; 3 Department of Nutrition and Integrative Physiology, University of Utah, Salt Lake City, Utah, USA; 4 Institute of Diabetes, Obesity, and Metabolism, University of Pennsylvania, Philadelphia, Pennsylvania, USA; 5 Department of Biomedical Sciences, University of Missouri, Columbia, Missouri, USA

**Keywords:** heart, diabetes, insulin, GRK2, islet, glucose, obesity

## Abstract

Cardiovascular diseases are a major comorbidity factor in patients with type 2 diabetes and a leading cause of death among them. Yet, mechanistically, how impairment in pancreatic islets alters cardiac function under different metabolic states remains largely unknown. Here, we investigate the role of the G-protein-coupled receptor kinase 2 (GRK2) in regulating islet adaptations to an obesogenic diet and its impact on myocardial function. Using a novel inducible β-cell-specific GRK2 knockout mouse model (βGRK2KO), we establish that loss of adult β-cell GRK2 delays metabolic islet maladaptation, protecting the heart against obesity-induced cardiac dysfunction. βGRK2KO are more insulin-sensitive than control mice and have improved cardiac function and myocardial morphology. Thus, genetic ablation of GRK2 in adult β-cells during an obesogenic diet play a cardioprotective role. This study prompts a novel therapeutic window for GRK2 intervention strategies for diabetic patients prone to cardiac dysfunction.

Obesity is a chronic metabolic syndrome affecting children and adults worldwide (1). Over the last decades, obesity has emerged as a leading global health concern amplified by dietary reliance on the consumption of high-calorie or high-fat foods and a more sedentary lifestyle (1). Obesity is a major risk factor for type 2 diabetes (T2D) and cardiovascular diseases (2, 3). A T2D diagnosis correlates with an increase of up to four times the likelihood of developing heart failure (HF) (4). Yet, the clinical toolbox for HF interventions is limited and, for the most part, not personalized to include diabetic/pre-diabetic considerations. The pancreas is essential for digestion and regulation of blood sugars. The myocardium has little to no energetic storage; thus, the heart heavily relies on circulating queues and nutrients to generate ATP and elicit contraction (5). Yet, the majority of studies have separately focused on the pancreas or the heart, with fewer studies aiming at understanding the cross-talk of these organs. This is partially due to the difficulty in models that can discern between variable rates of adiposity from dysfunctional islet biology and disease onset (6). Overall, these factors have restricted holistic understanding of spatiotemporal changes in the pancreas that impact metabolically active organs such as the heart. Nonetheless, there is a dire need to better understand pancreas-heart crosstalk and its dependency on metabolic factors.

T2D is a complex disease with progressive stages that ultimately integrate a whole-body response to increased circulating substrate availability (4, 7). The primary initial focal point of dysfunction is within the pancreatic islet of Langerhans, yet metabolic adaptations occur in energetically active peripheral tissues, including the heart. Hyperinsulinemia is strongly associated with T2D (8). The first stage of T2D progression involves higher overall rates of insulin secretion and increased acute glucose-stimulated insulin secretion (9, 10). The hypersecretory phase has been postulated to act as both adaptive (7) and maladaptive (11) before the escalation of insulin resistance in peripheral tissues (12, 13). Nevertheless, consensus exists on the limited capacity for islets to augment insulin secretion in response to metabolic challenges, ultimately leading to islet stress, exhaustion, and failure (7, 14, 15).

Pancreatic β-cells produce and secrete insulin in response to increased levels of circulating metabolites including glucose and fatty acids (14). This response is known to be fine-tuned by a variety of modalities including activation of various G-protein-coupled receptors (GPCRs). For instance, α2-adrenergic receptor (α2AR) is highly expressed in β-cells, coupling to Gαi, and reducing insulin secretion (16). Expression of α2AR has been implicated in modulating insulin secretion and diabetes progression (17). Clinical reports of α2AR genetic variants have provided an association with obesity and diabetes (18, 19). Although GPCRs, particularly α2AR, have been shown to regulate islet function, much less is known about receptor regulation in the β-cell (20, 21). GPCR kinase 2 (GRK2) phosphorylates activated GPCRs and canonically initiates receptor endocytosis, triggering receptors to recycle or degrade (22). We have shown that GRK2 is the major GRK isoform in the islet and loss of whole-pancreas GRK2 at the embryonic stage results in decreased insulin secretion, accelerated weight gain, and impaired cardiac function (23). The developmental impact of whole pancreas GRK2 knockout at the embryonic stage (23) is consistent with studies in global GRK2 knockout where embryonic lethality was reported over 30 years ago (24). GRK2 is known to participate in key developmental processes, and while intriguing, the pancreatic-specific model affects acinar and islet cellular function during vital developmental processes.

The specific role of GRK2 in regulating adult β-cell function and its impact on the heart remains largely understudied. Considering pancreatic development and contribution to endocrine and exocrine function, the goal of this study was to investigate and address the role of GRK2 in regulating adult β-cell function and, subsequently, cardiac biology in response to metabolic challenges using a Western diet model of high-fat high-sucrose (HFHS) diet. Our results show that adult β-cell GRK2 plays a significant role in modulating insulin secretory mechanisms, particularly during dietary metabolic challenges. Importantly, the results show that adult β-cell GRK2 does not alter adiposity rates but leads to a subsequent amelioration in muscle glucose uptake, cardiac tissue remodeling, and myocardial function.

## Results

### Generation of **β**GRK2KO and characterization of phenotype

**Figure 1:**
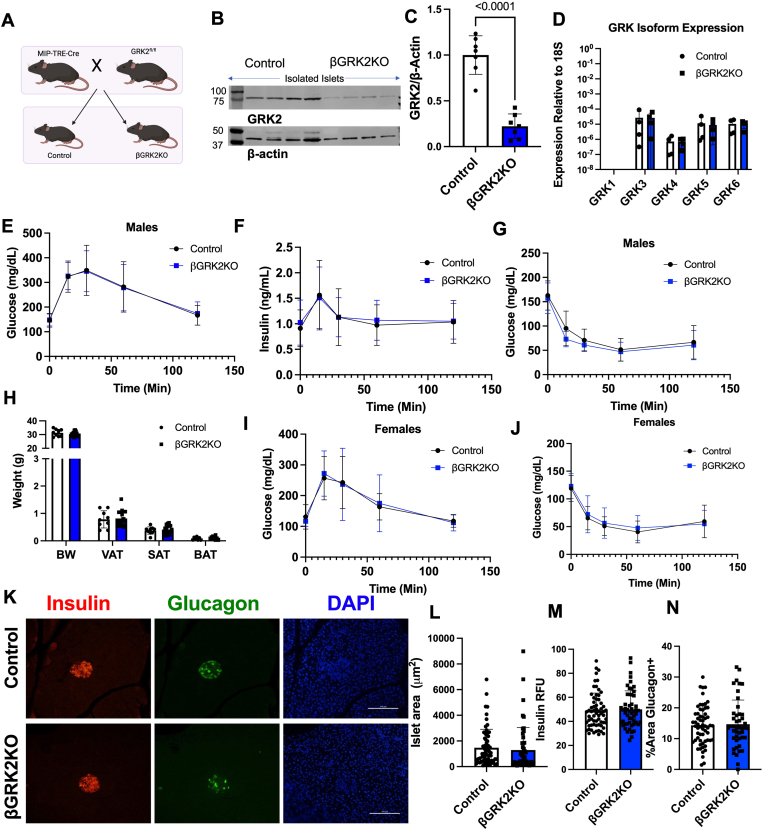
**Acute adult β-cell GRK2 knockout does not alter glycemic control in normal physiology**. *A*, graphical representation of novel transgenic knockout mouse line for inducible β-cell specific KO of GRK2. *B*, representative Western blot of GRK2 and loading control β-Actin using lysates of isolated pancreatic islets from control and βGRK2KO mice fed doxycycline chow. *C*, densitometry of Western blots for GRK2 using isolated islet lysate (n = 8 mice/group). *D*, TaqMan qPCR analysis of the expression of all GRK isoforms from male pancreatic islet RNA using 18S as an endogenous loading control. *E*, Glucose levels during OGTT of male animals on doxycycline (n = 33 control and 22 βGRK2KO mice). *F*, Insulin levels during OGTT (n = 27 control and 15 βGRK2KO mice). *G*, Insulin tolerance test of male mice fed doxycycline standard chow (n = 15 control and 11 βGRK2KO mice). *H*, terminal analysis of BW, BAT, VAT, and SAT in dox-treated animals. *I*, Glucose levels during OGTT of female mice exposed to doxycycline (n = 18 control and 13 βGRK2KO mice). *J*, Glucose levels during insulin tolerance testing of female mice, doxycycline diet (n = 22 control and 15 βGRK2KO mice). *K*, representative images of pancreatic islets for insulin and glucagon; scale bar is 100 μm. *L*, Islet area analysis. *M*, Insulin signal analysis; Relative Fluorescence Units (RFU). *N*, percent area stained positive for glucagon, islet quantification displayed in 5 separate animals per group for panels *L*–*N*. Two-tailed student’s t-tests were used in panels *C*, *D*, *L*, *M*, and *N*. AUC followed by *t* test was performed in panels *E*–*G*, *I*–*J*. Data are shown as mean ± SD.

Tamoxifen is known to suppress pancreatic islet cell proliferation (25), thus we generated a novel mouse model of an inducible β-cell GRK2 knockout utilizing a TET On-Cre system (Fig. 1*A*) (26). Inducible β-cell GRK2 homozygous knockout (βGRK2KO) exhibited approximately 80% decrease in islet GRK2 (Fig. 1, *B* and *C*). Considering that murine β-cells constitute 80% of islet cells (27, 28), this model achieves nearly complete GRK2 knockout in the desired cell type. This downregulation is not linked to alterations in other GRK isoforms (Fig. 1*D*).

**Figure 2:**
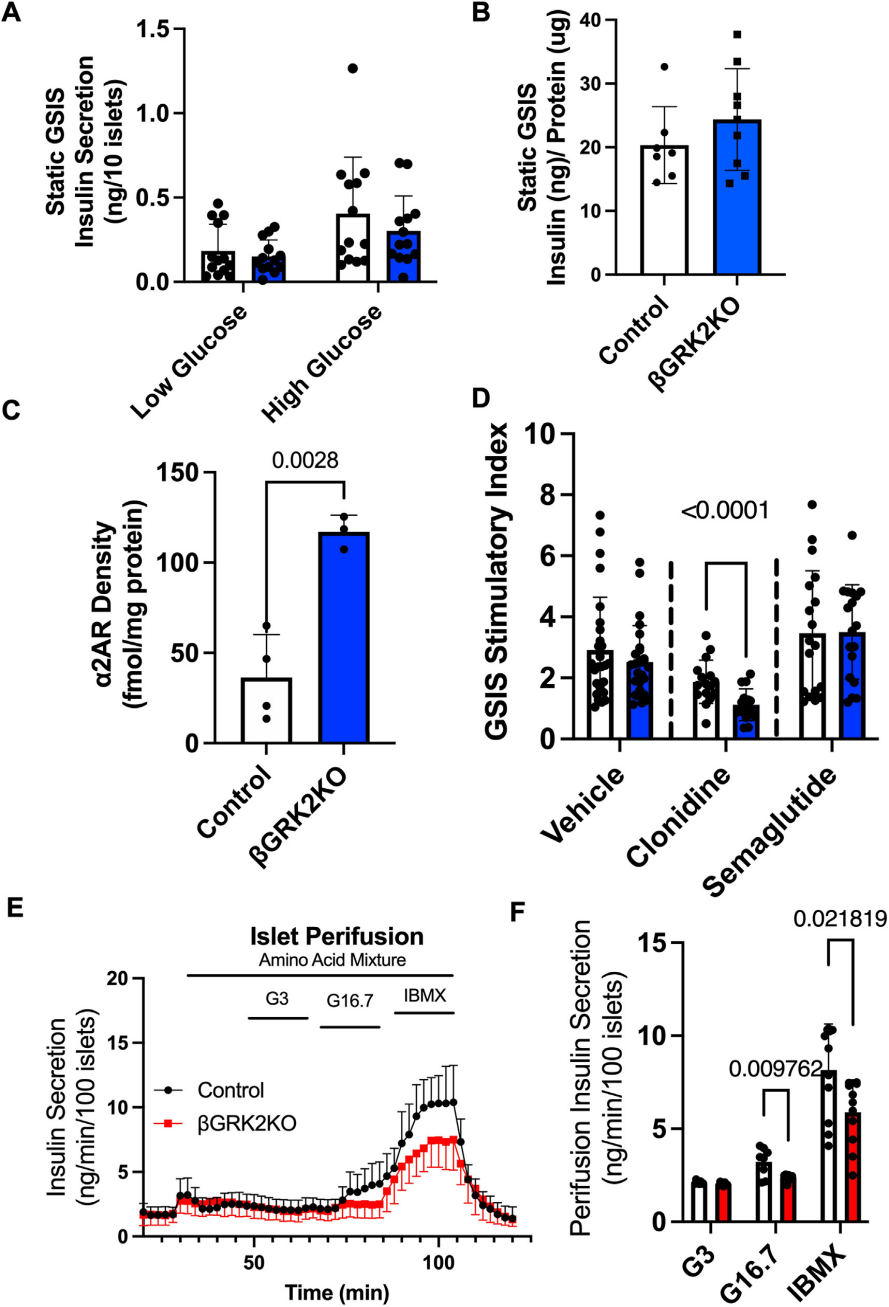
**βGRK2KO islets exhibit altered glucose response *ex vivo* and α2AR density**. *A*, Insulin secretion in a static media experiment. *B*, Insulin content in islets used for static insulin secretion experiments. *C*, α2-Adrenergic receptor density in isolated membranes from pancreatic islets in dox diet (n = 4 control and n = 3 βGRK2KO mice per group). *D*, stimulatory index of insulin secretion for islet pools treated with low glucose then high glucose with or without drug treatment (6 separate mice per group, islet pools displayed on graph), clonidine 100 nM and semaglutide 100 nM. *E,* Insulin secretion during islet perifusion using primary islets isolated from mice challenged *in vitro* (n = 4 mice/group). *F*, quantification of insulin secretion from each period of exposure in *E*. Two-tailed student’s t-tests were used for panels *A*–*D*, *F*. Data are shown as mean ± SD.

Adult β-cell GRK2 knockout did not alter glucose tolerance (Fig. 1*E*), insulin secretion (Fig. 1*F*), or insulin tolerance (Fig. 1*G*) when compared to control animals. No significant body weight or adiposity changes were observed when β-cell GRK2 was knocked out (Fig. 1*H*). These observations were similar in males and females (Fig. 1, *I*–*J*). At the islet level, islet size, insulin-positive cells, and glucagon-positive cells were not impacted by the loss of β-cell GRK2 in the adult stage (Fig. 1, *K*–*N*). Glucose-stimulated insulin secretion (GSIS) in βGRK2KO islets was not statistically different from control islets in static media containing low and high glucose levels (Fig. 2*A*), and total insulin content in βGRK2KO islets was comparable to control islets (Fig. 2*B*). However, plasma membrane α2AR expression in βGRK2KO islets was statistically increased when compared to control islets (Fig. 2*C*).

### GRK2 participates in **β**-cell adaptation to metabolic challenges *in vitro* and *in vivo*

Secreted insulin in βGRK2KO islets was similar to control islets (Fig. 2*D*); however, co-treatment with clonidine (α2AR agonist) elicited a statistically significant decrease in βGRK2KO islets insulin secretion compared to control islets (Fig. 2*D*). No statistical differences between genotypes were observed in islets stimulated with semaglutide, a GLP1-R agonist, on static GSIS (Fig. 2*D*). Clonidine and semaglutide concentrations were selected based on dose-response curves that exhibited maximal secretory responses. Perifusion studies in isolated islets cultured in elevated glucose prior to experimentation revealed a statistically significant decrease in insulin secretion in βGRK2KO islets compared to control islets (Fig. 2, *E* and *F*), suggesting a dependence between islet GRK2 function and secretion of insulin during metabolic challenges.

**Figure 3:**
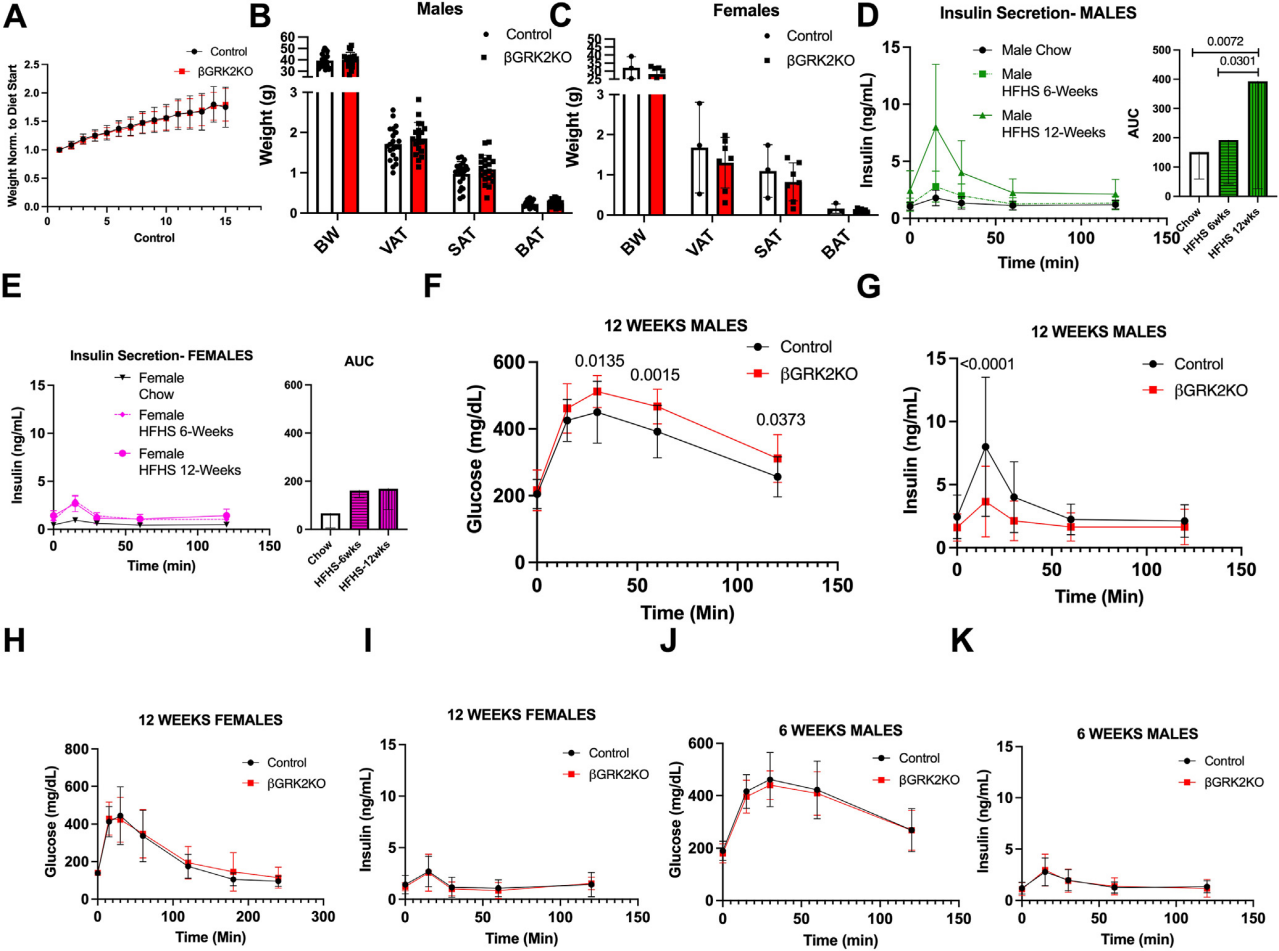
**Inducible GRK2 KO in β-cells predisposes mice to decreased HFHS-induced glucose tolerance with lower insulin secretion after 12 weeks on diet**. *A*, body weight analysis during HFHS diet exposure (n = 21 animals/group). *B*, terminal body weight and adipose tissue measurements at 15 weeks of exposure to HFHS diet in male mice. *C*, terminal body weight and adipose tissue measurements at 15 weeks of exposure to HFHS diet in female mice. *D*, control male mice adaptation to diet over time (n = 15–19 mice/group), AUC plots are shown next to trace. *E*, control female mice adaptation to diet over time (n = 3–18 mice/group), AUC plots are shown next to trace. *F*, Glucose exposure during OGTT was performed after 12 weeks on diet (n = 21 animals/group). *G*, serum insulin concentration during the same OGTT (n = 15 control and n = 19 βGRK2KO). *H*, Glucose levels during OGTT in female mice fed HFHS diet for 12 weeks (n = 4 control and 14 βGRK2KO mice). *I*, Insulin levels during OGTT in female mice fed HFHS diet for 12 weeks (n = 3 control and 7 βGRK2KO mice). *J*, Glucose levels during OGTT in male mice fed HFHS diet for 6 weeks (n = 21 mice/group). *K*, Insulin levels during OGTT in male mice fed HFHS diet for 6 weeks (n = 19 control and 18 βGRK2KO mice/group). Two-tailed t-tests were performed in panels *B* and *C*. One-way ANOVA was performed on AUC plots in panels *D* and *E*. A 2-way ANOVA with multiple comparisons test was performed in panels *F*–*K*. Data are shown as mean ± SD.

**Figure 4:**
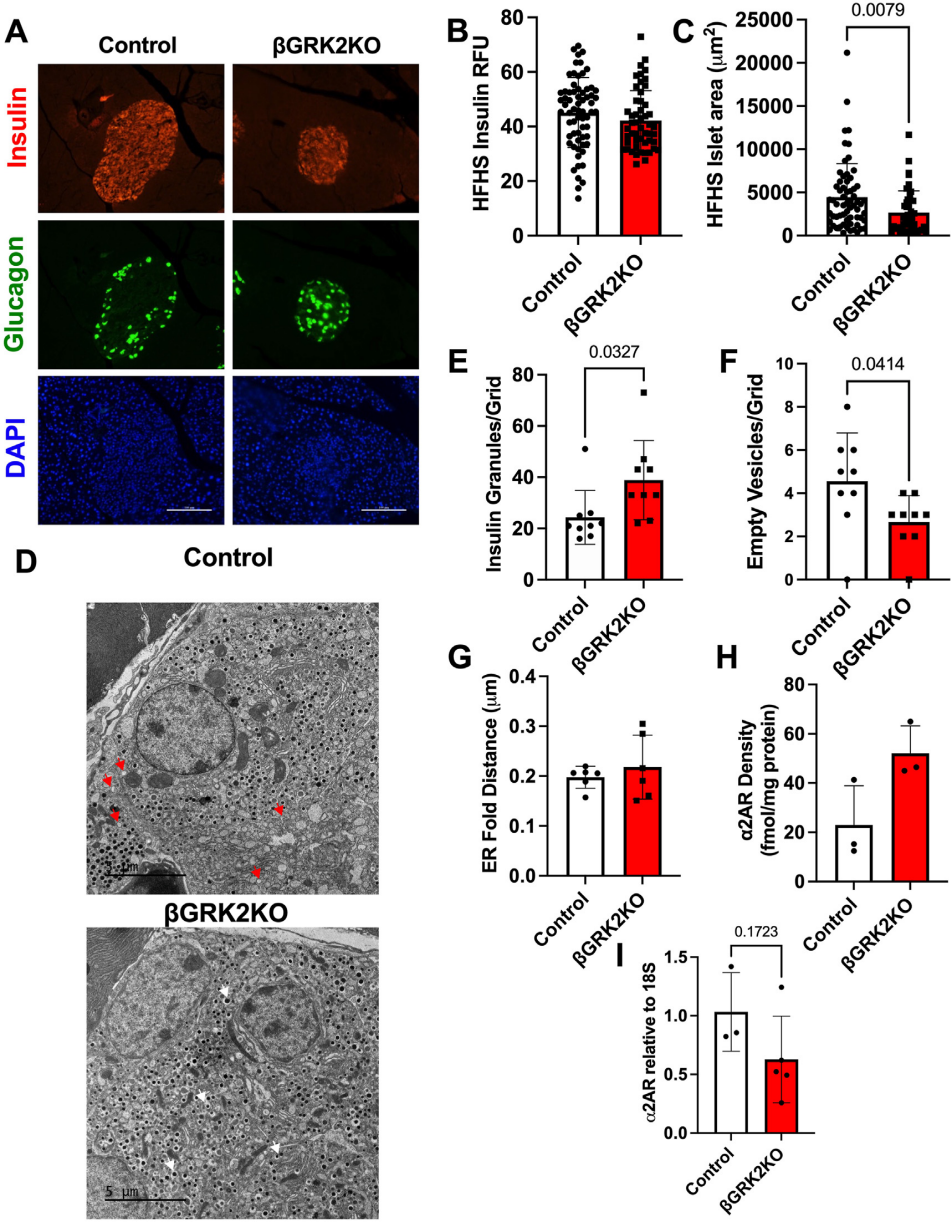
**Adaptive insulin alterations to diet in βGRK2KO compared to control islets**. *A*, Representative images of insulin and glucagon in pancreatic islets from male mice fed HFHS for 15 weeks; scale bar is 100 μm. *B*, Insulin RFU analysis (islet quantification displayed of six mice/group). *C*, Islet area analysis of HFHS islets (n = same as *B*). *D*, representative electron micrographs of β-cells at 1500X magnification (scale bar is 5 μm); red arrowhead-examples of empty vesicles, white arrowheads-examples of insulin granules. *E*, quantification of insulin granules, grid analysis from three mice/group. *F*, quantification of empty vesicles in β-cells (Same n as in *E*). *G*, average distance from the nucleus to the folds of endoplasmic reticulum (n = 3mice/group). *H*, α2AR density in isolated membranes from pancreatic islets under HFHS (n = 3 mice/group). *I*, α2AR mRNA levels were measured by qRTPCR in isolated islets subjected to HFHS; 18s was used as housekeeping gene for normalization (n = 3 control mice and 5 βGRK2KO). Two-tailed student’s *t* test was performed in panels *B*–*C*, *E*–*I*. Data are shown as mean ± SD.

To determine the role of adult β-cell GRK2 in islet adaptation, we implemented a HFHS dietary regimen to model Western diets known to necessitate islet adaptation. βGRK2KO mice gained weight at a similar rate as control animals (Fig. 3*A*). Body weight and adiposity development in HFHS were not statistically different among the groups in both males and females (Fig. 3, *B* and *C*). A time-course analysis of insulin secretion during OGTT in control male mice (Fig. 3*D*) showed a significant increase in insulin secretion at 12-weeks in response to HFHS exposure. Following the same timeline, this adaptation was not observed in female control mice (Fig. 3*E*). These sex differences culminated at 12 weeks post-diet, where βGRK2KO male mice exhibited a moderate but significant elevation in glucose levels during OGTT when compared to control mice (Fig. 3*F*). This alteration was linked to differential insulin secretion in βGRK2KO mice (Fig. 3*G*). Using the same timeline, at 12 weeks of HFHS exposure, βGRK2KO female mice presented with comparable glucose tolerance and insulin secretion to its control mice counterparts (Fig. 3, *H* and *I*). This latter observation is possibly due to a limited or delayed islet adaptation induced by HFHS in female mice when compared to males (Fig. 3, *D* and *E*). No differences in glucose tolerance and insulin secretion between control and βGRK2KO males were observed at 6 weeks of HFHS (Fig. 3, *J*–*K*). As such, hereafter, the study focused on male mice post-12 weeks of HFHS diet, where a phenotype was observed. Analysis at the islet level (Fig. 4*A*) showed that loss of β-cell GRK2 did not significantly alter insulin (Fig. 4*B*), but significantly diminished islet expansion in response to HFHS diet (Fig. 4*C*). Electron microscopy analysis of islets from mice in the HFHS diet regimen showed an increase in insulin-containing granules and fewer empty-vesicle granules in βGRK2KO β-cells compared to control β-cells (Fig. 4, *D*–*F*). Average distance in ER fold was comparable in both groups (Fig. 4*G*). A trend toward increases in α2AR was observed in islets from βGRK2KO after HFHS (Fig. 4*H*). Expression of α2AR at the message level was not statistically different from control islets (Fig. 4*I*).

**Figure 5:**
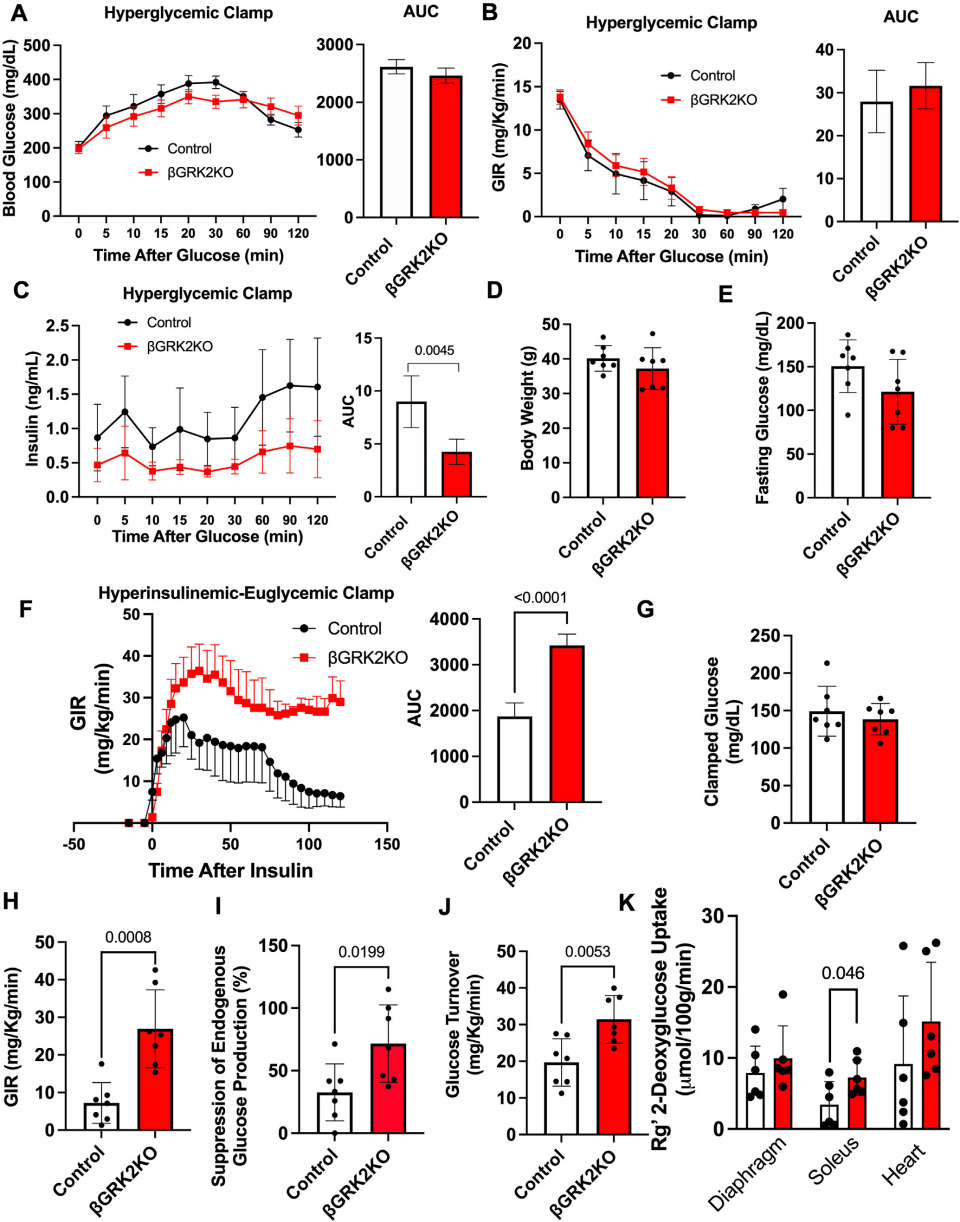
**Altered islet and peripheral glucose uptake in βGRK2KO accessed by clamp studies**. *A*, Blood glucose levels during hyperglycemic clamps, AUC is shown on the right of the trace. *B*, Glucose infusion rates (GIR) during hyperglycemic clamps, AUC is shown on the right of the trace. *C*, blood insulin levels during hyperglycemic clamps, AUC is shown on the right of the trace (n = 7 mice/ group). *D*, body weight of animals before hyperinsulinemic clamps. *E*, fasting glucose levels of animals before hyperinsulinemic clamps. *F*, Glucose infusion rates during hyperinsulinemic-euglycemic clamp performed on male mice on HFHS (n = 7 mice/group), AUC for the clamp is shown to the right of the trace. *G*, clamped glucose during hyperinsulinemic-euglycemic clamps. *H*, Glucose infusion rates (GIR) for clamped state in *F*. *I*, suppression of endogenous glucose production. *J*, Glucose turnover for experiments in *F.K*, 2-Deoxyglucose uptake in organs from *F*. Two-tailed student’s t-tests were performed in all plots in this figure. Data are shown as mean ± SD.

To confirm the effects on insulin secretion in the context of the HFHS diet regimen, we performed hyperglycemic clamps to assess insulin secretion (Fig. 5, *A*–*C*). We achieved a similar degree of hyperglycemia in βGRK2KO and control mice (Fig. 5*A*) with comparable glucose infusion rates (GIR; Fig. 5*B*), however, insulin secretion in response to glucose was substantially reduced in βGRK2KO mice (Fig. 5*C*). As βGRK2KO under HFHS showed moderate glucose control despite substantially lower circulating insulin levels, we examined insulin sensitivity in these mice. Total body weight and fasting glucose were comparable between control and βGRK2KO mice (Fig. 5, *D* and *E*). Indeed, the insulin sensitivity index calculated after HFHS suggested that βGRK2KO mice are more insulin-sensitive than control mice. Hyperinsulinemic-euglycemic clamps in HFHS animals showed a statistically significant increase in whole-body insulin sensitivity in βGRK2KO mice compared to control animals (Fig. 5*F*), confirming increased insulin sensitivity in peripheral tissues. Clamped glucose levels were comparable among the groups (Fig. 5*G*). GIR for the clamped state was enhanced in the βGRK2KO mice compared to control animals (Fig. 5*H*). Insulin-mediated suppression of endogenous glucose production was increased (Fig. 5*I*). Glucose-turnover, indicative of increased glucose uptake in muscle, was elevated in βGRK2KO mice compared to control mice (Fig. 5*J*). Enhanced uptake of 2-deoxyglucose was observed in the soleus muscle with a trend towards an increase in the heart (Fig. 5*K*). ^3^H-glucose kinetics revealed that both liver and muscle contribute to improved insulin sensitivity observed in the βGRK2KO mice.

### Loss of **β**-cell GRK2 in obesity protects cardiac structure and function

**Figure 6:**
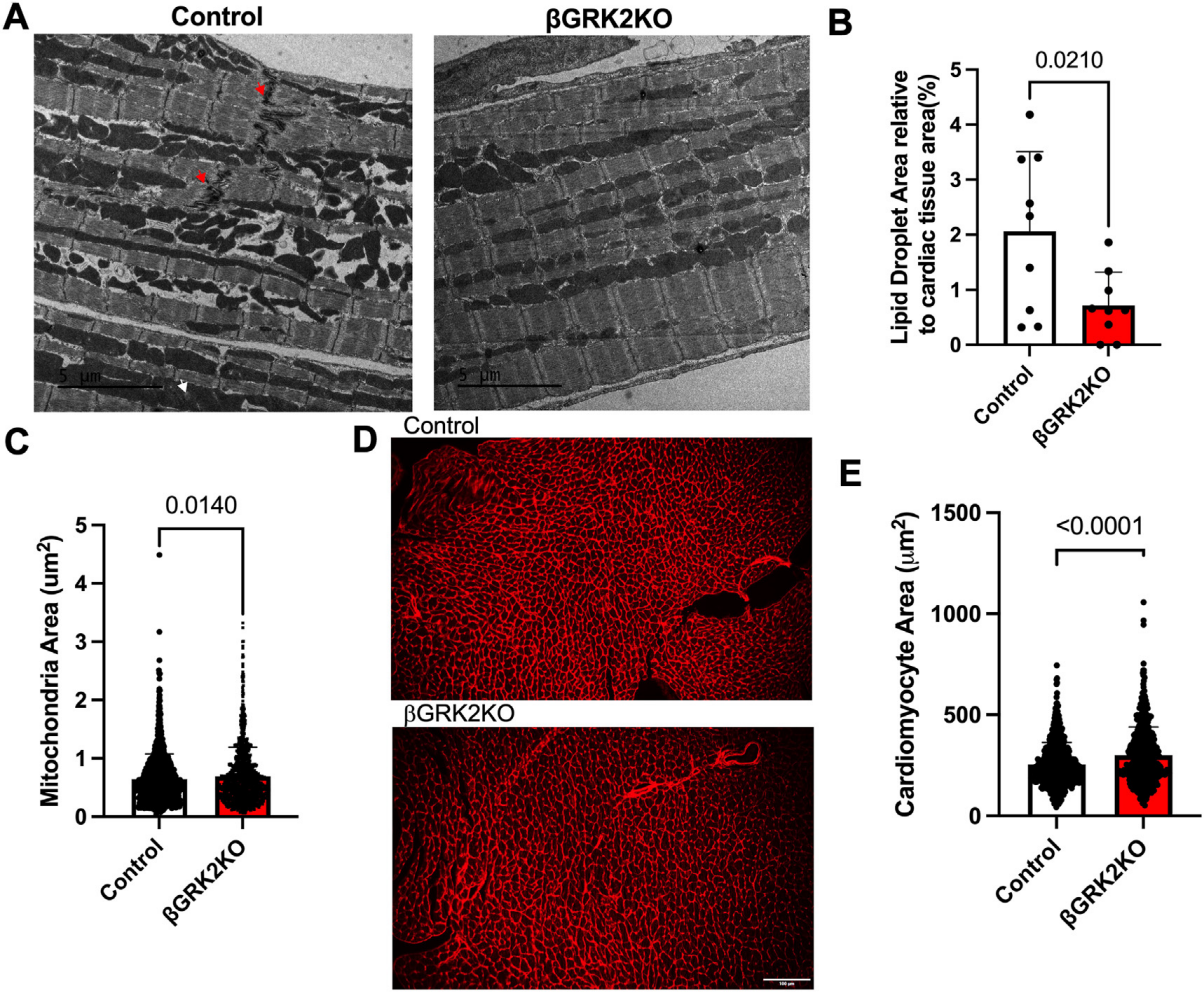
**Improved cardiac structure in response to HFHS diet regimen in βGRK2KO mice**. *A*, representative electron micrographs from hearts exposed to HFHS, white arrowheads represent a lipid droplet, red arrowhead indicate intercalated discs, scale bar is 5 μm. *B*, lipid droplet area analysis, displayed are images analyzed from three mice/group. *C*, Cardiac mitochondrial area, displayed are individual mitochondria analysis from three mice/group. *D*, representative images of cardiac tissue stained with WGA, scale bar is 100 μm. *E*, analysis of cross-sectional myocyte area from images in *D* (n = 7 mice/group). Two-tailed student’s *t* test was used in panels *B*, *C*, and *D*. Data are shown as mean ± SD.

**Figure 7:**
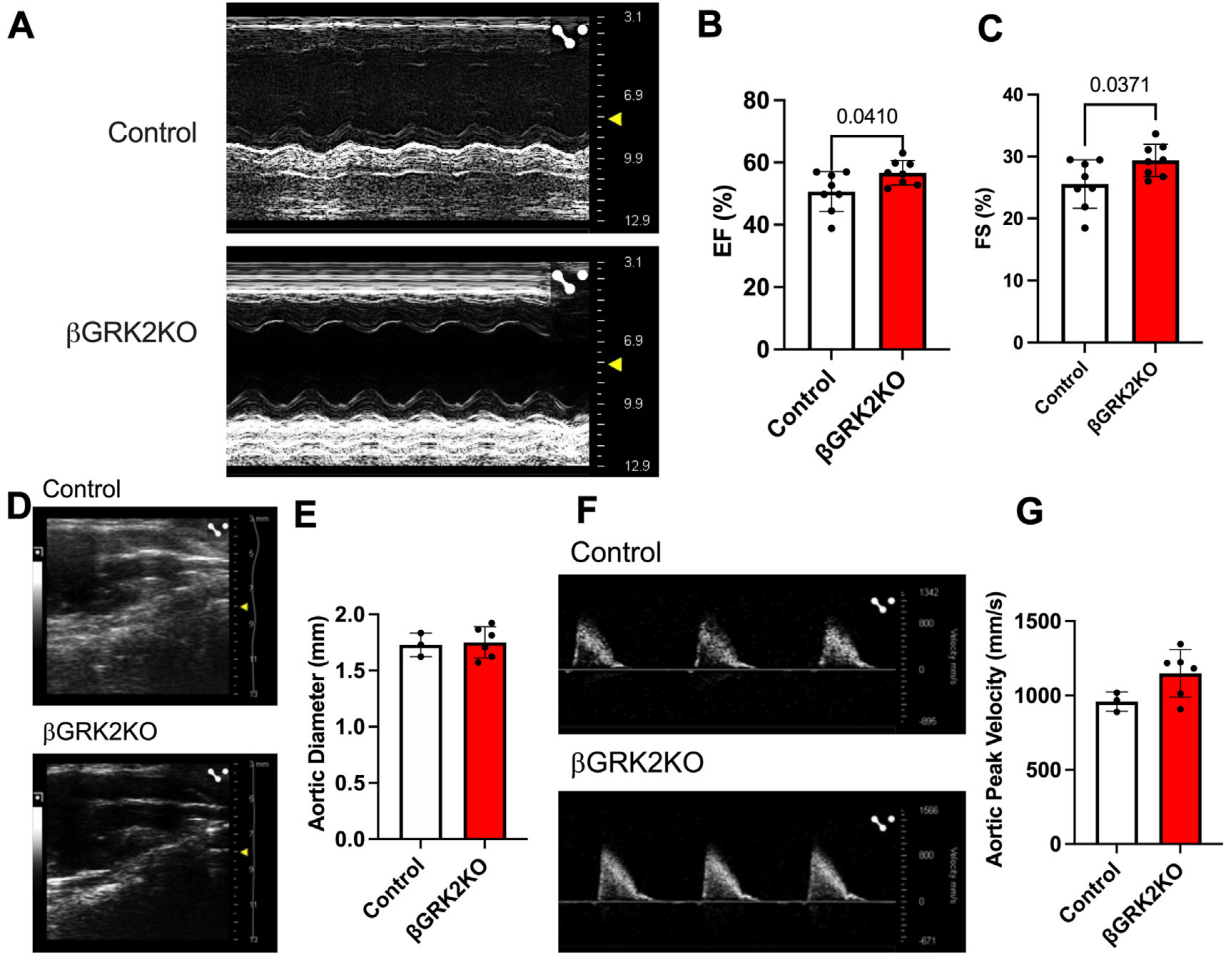
**Cardiac function is superior in βGRK2KO mice exposed to HFHS when compared to control mice**. *A,* representative M-mode echocardiography acquisition. *B*, cardiac ejection fraction quantification. *C*, fractional shortening analysis. *D,* representative aortic diameter image. *E*, aortic diameter analysis. *F*, Representative aortic peak velocity. *G*, Quantification of aortic peak velocity. Displayed are individual animals. *t* test was performed in panels *B*, *C*, *E* and *G*. Data are shown as mean ± SD.

Electron microscopy analysis of HFHS hearts showed tissue alterations at the structural level (Fig. 6*A*) in control hearts that were not observed in βGRK2KO hearts. Lipid droplet accumulation was decreased in βGRK2KO hearts (Fig. 6*B*) with a moderate but statistically significant increase in myocardial mitochondrial area (Fig. 6*C*). Cardiomyocyte cell area was increased in βGRK2KO compared to control mice exposed to HFHS (Fig. 6, *D* and *E*). Functionally, HFHS βGRK2KO hearts exhibited improved cardiac function measured by ejection fraction and fractional shortening (Fig. 7, *A*–*C*), with no alterations in aortic diameter (Fig. 7, *D* and *E*), nor aortic peak velocity (Fig. 7, *F* and *G*).

## Discussion

We have shown that the embryonic loss of GRK2 in the entire pancreas leads to glucose intolerance, increased body weight, and reduction in cardiac function (23). The latter study was confounded by the developmental roles of GRK2 in the pancreas, both at the endocrine and exocrine levels, as well as a negative myocardial nutritional impact necessary for proper organ development. The pancreatic GRK2 knockout findings align with the global GRK2 knockout mouse model, which includes embryonic lethality partially due to hypoplasia of both right and left ventricles (24). Using heterozygous animals from the global GRK2 model, recent studies have suggested a GLP1R-mediated potentiation of early insulin release in global GRK2 heterozygous animals compared to control mice (29). The use of the heterozygous genotype from the global GRK2 knockout model is confounded by the impact of GRK2 expression in all organs, potential compensation of other GRKs, and unknown impact on islet function during development. To understand the role of GRK2 in the adult mature β-cell, we developed a model to specifically and inducibly knockout GRK2 in adult β-cells. Loss of GRK2 in adult β-cells is significant (Fig. 1, *B* and *C*), and the magnitude of knockout is close to totality given the known β-cell-type composition in murine islets (27, 28). Loss of β-cell GRK2 *per se* does not acutely impact islet function as glucose tolerance, insulin secretion, and insulin tolerance are comparable between genotypes in both sexes (Fig. 1 and Fig 2, *A* and *B*). Remarkably, loss of β-cell GRK2 led to an upregulation of α2AR expression at the plasma membrane (Fig. 2*C*), potentially queuing these cells for altered islet remodeling responses and context-specific insulin secretion suppression (Fig. 2*D*). As GRK2 regulates α2AR (30, 31), this increase in baseline α2AR at the plasma membrane is relevant as this receptor is known to couple to Gαi and exert an inhibitory effect on islet insulin secretion (32). The increase in baseline α2AR may be particularly relevant during HF, (with increased circulating catecholamines) or during metabolic challenge/syndrome.

Notably, perifusion experiments of βGRK2KO islets cultured in high glucose levels led to diminished insulin secretion in response to high glucose stimulation, suggesting a propensity for altered secretion regulated by islet GRK2 (Fig. 2, *E* and *F*). Interestingly, studies in obese individuals without hypertension or diabetes found that insulin hypersecretion was more prevalent than insulin resistance (11) and proposed to precede insulin resistance. The RISC study supports this concept, where greater insulin secretory responses were observed independently of insulin sensitivity, hence supporting a primary defect rather than compensation to insulin resistance (33, 34).

In this study, metabolic challenge induced by the introduction of HFHS led to a male-predisposition phenotype in βGRK2KO mice when compared to controls with a moderate but significant decrease in glucose tolerance that was linked to decreased insulin secretion during OGTT (Fig. 3, *F* and *G*) or during hyperglycemic clamps (Fig. 5, *A*–*C*). These observations may be delayed in females (Fig. 3, *D* and *E*). The more adaptive insulin response in βGRK2KO animals was linked to increased insulin sensitivity in peripheral tissues as measured by hyperinsulinemic-euglycemic clamps (Fig. 5*F*), improved myocardial ultrastructure (Fig. 6, *A*–*C*), and cardiac function (Fig. 7*A*). The observation that there is increased expression of α2AR at the plasma membrane of islets without HFHS diet (Fig. 2*B*) may play a significant role in protecting islet health during distinct metabolic regimens. Loss of GRK2 does not significantly alter α2AR mRNA (Fig. 4*I*) but it does change protein at the plasma membrane (Figs. 2*C* and 4*H*). This is in agreement with the role of GRK2 in receptor turnover (22). In studies using Min6 cells, a cell line model of islets, GRK2 knockdown led to diminished extracellular calcium entry *via* Gαi/o signaling (23). Islet extracellular calcium entry is mediated by the L-type calcium channel. Clinically and experimentally, verapamil, a L-type calcium blocker, protects islets and prolongs islet health (35, 36). The implication of safeguarding this pathway in the islet to cardiac health is largely understudied and further supported by this work.

HFHS hearts from the βGRK2KO mice have slightly larger cardiomyocyte size with improved cardiac ejection fraction when compared to control mice. This could be partially attributed to the improvement in insulin sensitivity (Fig. 5*F*) which includes increased heart glucose uptake (Fig. 5*K*), decreased cardiac lipid droplet content (Fig. 6*B*), increased mitochondrial area (Fig. 6*C*), and maintenance of myocardial ultrastructural tissue arrangement (Fig. 6*A*). Importantly, loss of β-cell GRK2 did not impact aortic diameter, or aortic peak velocity (Fig. 7, *D*–*G*), suggesting comparable aortic physiology. This data agrees with studies in mice showing that normalizing circulating insulin enhances insulin sensitivity and extends lifespan (37). The role of islet GRK2 in cardiac remodeling during metabolic challenges is important, and we hypothesize that it participates in T2D-driven cardiac dysfunction. This data supports the notion that having a more regulated glucose-mediated hyper insulin secretion phase prolongs islet health and increases the reserve of insulin responses.

Notably, GRK2 myocardial inhibition strategies are beneficial in various animal models of HF (38, 39, 40), and currently, myocardial GRK2 inhibition is being pursued by various groups as a mode of HF treatment (41, 42). The CARE-AMI clinical trial using paroxetine, an SSRI inhibitor that can also inhibit GRK2 (43), did not show improvements in LVEF recovery in patients 12-weeks post infarction (44) or at 1 year in patients with STEMI (45). This latter study is limited by participant numbers and does not subcategorize the results to include information on pre-diabetic/diabetic conditions. Our previous study using the whole pancreas GRK2 knockout alongside this study suggests an important developmental role for β-cell GRK2, as well as a role in islet adaptation to metabolic challenges that are not linked to differential accumulation of adipose tissue and/or BMI. We propose that important overall clinical considerations are relevant when investigating GRK2 inhibitory therapy as a mode of cardiac protection. In particular, we propose that GRK2 inhibitory approaches are beneficial to a subset of prediabetic/diabetic patients prone to cardiac dysfunction, especially where glycemic control can be established by non-insulin therapy such as diet, exercise, and metformin usage. Current clinical trials may be masking the true potential of GRK2 inhibition strategies in HF, as glucose control and insulin levels are not often a criteria for diversification.

## Experimental procedures

### Generation of **β**GRK2KO mouse model

All animal experimental procedures were approved by the Institutional Animal Care and Use Committee at Drexel University College of Medicine and the University of Alabama at Birmingham, and all methods were performed under the relevant guidelines and regulations. The βGRK2KO mouse model was constructed by crossing the Tre-Cre containing the MIP-rtTA sequence (26) (kind gift of Philipp Scherer) with the GRK2 Flox line (23). All mice were genotyped accordingly before enlistment in the study.

### Dietary regimen

Regular chow (D09050202) was obtained from Research Diets containing 200 mg of doxycycline per 4057 kcal. HFHS diet consisted of 40% of calories from fat and 40% of calories from sucrose and was matched to the standard chow diet for doxycycline content normalized to caloric value (D21020902). Both diets were initiated in mice between 6 and 8 weeks of age. For regular chow studies, 4 to 6 weeks of diet exposure defined the window of study unless otherwise noted. For HFHS studies, a longitudinal study of up to 15 weeks of exposure was conducted.

### Pancreatic islet isolation

Islet isolation was performed as previously described (46). The common bile duct was ligated before animal sacrifice by exsanguination *via* cardiac dissection. The pancreas was perfused with collagenase and digested. Islets were hand-picked using a Leica S9i Stereoscope and cultured for 1 day in RPMI 1640 with 10% FBS, 1% penicillin/streptomycin, 3 mM glucose, and 2 mM glutamine.

### RNA isolation and qPCR

RNA was extracted from pancreatic islets *via* Purelink RNA mini-kit (Ambion) according to manufacturer’s protocol. RNA to cDNA conversion was performed using High-Capacity cDNA conversion kit (ThermoFisher) according to manufacturer’s protocol and using reaction sizes between 250 ng and 1 μg of RNA input. qPCR reactions were performed with TaqMan reagents for GRK isotypes using 12.5 ng cDNA/reaction. All reactions were performed in triplicate using QuantStudio 7. ΔCt values were calculated using 18S as the housekeeping gene. GRK1 (Mm01220712_m1), GRK2 (Mn00804778_m1), GRK3 (Mm00622042_m1), GRK4 (Mm01213690_m1), GRK5 (Mm00517039_m1), GRK6 (Mm00442425_m1), 18s (Mm02619580_g1). For α2AR mRNA, SYBR reactions followed manufacturer’s protocol. Primers used were: mADRA2A Fwd (CTGGCTGAGATCATGTGACTAC), mADRA2A Rev (CCTTCCACAGTCTGCCTAAA), 18s FOR (GTAACCCGTTGAACCCCATT), 18s REV (CCATCCAATCGGTAGTAGCG).

### Preparation of lysates and Western blot analysis

Western blots were performed as previously described (47). Isolated islets were lysed in RIPA buffer. Protein quantification was performed using BCA (Pierce) and 30 μg of protein was loaded onto tris–glycine gels. Primary antibodies were incubated overnight at 4 °C, washed three times in PBS with 1% Tween-20, incubated with secondary antibodies for 1 h at room temperature, and washed three more times. Imaging was performed using a LI-COR Odyssey Fc and analysis was performed off-line using Image Studio. Primary Antibodies used were: GRK2 Sigma G0296 and β-actin Santa Cruz 47778. Specificity of GRK2 antibody was previously conducted using genetic-mouse models with overexpression of GRK2 (48) and GRK2 knockdown (49).

### Oral glucose tolerance test

OGTT was performed as we have published elsewhere (50); fasting was performed using new clean cages to limit coprophagy. These mice were then given oral gavage glucose and blood glucose was measured by a glucometer at 0-, 15-, 30-, 60-, and 120-min time points. Serum was utilized for insulin measurements using an Ultra-Sensitive Mouse Insulin ELISA kit (Crystal Chem). For HFHS mice, additional glucose measurements were taken at 180- and 240-min.

### Insulin tolerance test

Mice were fasted for 2 h in clean cages at the same time of day to limit circadian variation. Intraperitoneal injections of insulin were given to mice at 1 unit/Kg and blood glucose was measured at 0-, 15-, 30-, 60-, and 120-min time points. Glucose was measured using a glucometer.

### Islet perifusion and insulin quantification

Pancreatic islets were isolated from mice and experiments were performed as reported elsewhere (51). Briefly, islets were cultured for 2 to 3 days in medium (RPMI 1640 containing 10 mM glucose, 10% FBS, and 1% penicillin/streptomycin); islets were size-matched and placed into perifusion chambers (100 islets/chamber) with Bio-Gel P-4 media (Bio-Rad) to immobilize them in an automated perifusion system (Biorep Perifusion System). All compounds used were dissolved in perifusion buffer (composition in mM: 125 NaCl, 5.9 KCl, 2.56 CaCl_2_, 1.2 MgCl_2_, 25 HEPES, and 0.1% BSA, pH 7.4). A peristaltic pump pushed reagents continuously into the islet-containing chambers. Perfusates were collected in ice-cold 96-well plates for further analysis after equilibration for 48 min in either three- or 12-mM glucose. Stimulation used contained 3.5 mM amino acid mixture, 3 mM glucose, 16.7 mM glucose, and 100 μM IBMX were sequentially flowed through the islet population and solution fractions were collected in 2-min increments. Insulin content in these fractions was detected by radioimmunoassay.

### Static media glucose-stimulated insulin secretion experiments

Static media glucose-stimulated insulin secretions experiments were performed similarly to published results (51). Pancreatic islets were isolated from mice and cultured overnight. Islets were washed with working HBSS (51) (HBSS containing a mixture of amino acids and 3 mM glucose). Islets were then hand-picked and deposited in groups of 10 size-matched into a 96-well microplate. These islets were allowed to equilibrate in working HBSS for 30 min at 37˚C and 5% CO_2_. The supernatant was removed and fresh working HBSS was introduced. The supernatant was collected and immediately flash-frozen. This supernatant was defined as the low glucose insulin secretion. Islets were then incubated with working HBSS, with or without clonidine (100 nM) or semaglutide (100 nM) and stimulated with glucose. Islet supernatant was harvested and flash-frozen. Islets were then collected and lysed with RIPA. Insulin concentrations from supernatant and lysate were determined by HTRF ultra-sensitive insulin assay using a PerkinElmer Envision Excite plate reader.

### Tissue fixation and immunofluorescence

Organs were fixed in 10% formalin overnight, embedded in paraffin, and sectioned at 10 μm thickness. Antigen retrieval was performed as published (52). Primary antibody incubation was performed overnight followed by three washes in PBS with 1% tween. Secondary antibody incubation was for 1 h at room temperature followed by three washes in PBS with 1% tween and mounting with DAPI. Imaging was performed using a Nikon Digital Sight Ri1 camera equipped on a Nikon Eclipse 50i microscope. Quantification was performed using ImageJ. Antibodies used were: Insulin Abcam ab7842, Glucagon Abcam ab92517, anti-guinea pig Cy3 Jackson Immuno Research 706-165 to 148, Anti AlexaFluor 488 Abcam ab150077. Insulin and glucagon marked β- and α-cell respectively. No signal was observed in acinar cells.

### Radioligand binding studies

Binding studies were performed as reported elsewhere (47). Briefly, membrane preparations were prepared from pancreatic islets as previously described (47). In brief, cells were lysed in ice-cold lysis buffer (25 mM Tris, pH 7.4, 5 mM EDTA, 1 μg/ml aprotinin, 1 μg/ml leupeptin) and centrifuged at 1000 x g for 5 min at 4°C. The supernatant was centrifuged at 30,000 x g and the crude membrane pellet was resuspended in lysis buffer containing 10% glycerol and stored at −80°C until use. Radioligand binding was performed using crude islet membranes as previously described (53). In brief, the density of α2-adrenergic receptors on membranes was determined by saturation radioligand binding. Reactions were performed by incubating membranes with 1 nM of 3H-RX821002 (PerkinElmer). Incubations were performed in a 250 μl total volume of binding buffer (75 mM Tris pH 7.4, 2 mM EDTA, 12.5 mM MgCl2, 1 μg/ml aprotinin, 1 μg/ml leupeptin) in the presence or absence of the α2AR antagonist RS79948 to determine specific binding. Reactions were allowed to equilibrate at 37°C for 1h before filtering through a glass fiber filter (Whatman GF/C; Brandel). Filters were washed with ice-cold buffer (10 mM Tris, pH 7.4, 10 mM EDTA) to remove unbound drug. The amount of total and non-specific radiolabeled RX821002 binding was determined using a liquid scintillation counter (PerkinElmer) and the specific binding (receptor density) was normalized to the amount of membrane protein.

### Hyperglycemic and hyperinsulinemic-euglycemic clamps

Hyperinsulinemic-euglycemic clamps were performed as described previously (54). Briefly, unrestrained mice were able to move freely while being continuously infused with insulin (2 mU/kg per min) and a variable infusion of 50% dextrose to allow for steady-state blood glucose of ≈120 mg/dl. Constant infusion of ^3^H-glucose throughout the experiment and for 90 min before the clamp allowed for the quantification of glucose infusion rate and uptake. At the end of a 2-h clamp, ^14^C-2-deoxyglucose (13 μCi per mouse) was administered during steady-state conditions. Hyperglycemic clamps were performed as in (55).

### Electron microscopy

Tissue preparation and processing were performed as previously described (47). Images were processed using FIJI/ImageJ to measure β-cell vesicle content, empty vesicle content, endoplasmic reticulum morphology, mitochondrial content, lipid droplet content, sarcomeric length, and intercalated disc morphology. Insulin granule content was measured using a percentage of volume relative to the β-cell area using a 260-grid point. The same grid was used to quantify empty vesicles. The maturity of vesicles was analyzed using the same parameters as published (56). Endoplasmic reticulum morphology was assessed by investigating the distance per endoplasmic reticulum fold. Mitochondria and lipid droplets were analyzed per unit area of myocyte as done (57). Sarcomeric length and intercalated disc measurements were calculated using Z-disc distances and convolution indexes as previously published (58).

### Wheat germ agglutinin membrane stain

Parrafin-embedded cardiac sections were deparaffinized and stained with wheat germ agglutinin (WGA Biotium) as published elsewhere (59). Briefly, sections were stained for WGA and mounted. Images were acquired using an Olympus fluorescence microscope BX43 F with a 20X objective using CellSens Standard version 3.2. Images were quantified using Image J software.

### Cardiac functional assessment by echocardiography

Echocardiography was performed as previously published (60) using the Vevo 2100 system. Mice were anesthetized using 2.5% isoflurane, and M-mode measurements were obtained at the level of the papillary muscles. B-mode was taken for aortic diameter and color Doppler was used for velocity measurements. Quantification was performed using VisualSonics software.

### Statistical analysis

All males and females were analyzed as indicated. All data are presented as mean ± SD. Statistical analyses were performed using Prism (GraphPad Software version 10). Statistical analyses were performed using two-sided unpaired t-tests (comparisons between two groups), one-way ANOVA with Tukey’s *post hoc* tests (for three groups or more), or two-way ANOVA for the main effect between control and KO groups with Sidak multiple comparisons tests. For perifusion assays and clamps, area under the curve (AUC) was analyzed when comparing more than two conditions. A *p*-value of less than 0.05 was considered statistically significant.

## Data availability

Data sets generated and analyzed in this manuscript are available upon request. Resource sharing is available upon request and MTAs.

## Conflict of interest

The authors declare that they have no conflicts of interest with the contents of this article.
